# Future temperature-related mortality in Latin American cities under climate change and population scenarios

**DOI:** 10.1016/j.envint.2025.109694

**Published:** 2025-07-23

**Authors:** Maryia Bakhtsiyarava, Josiah L. Kephart, Brisa N. Sánchez, M.V.S. Ramarao, Saravanan Arunachalam, Nelson Gouveia, Iryna Dronova, Leah H. Schinasi, Usama Bilal, Waleska T. Caiaffa, Andrea Jaffe, Ana V. Diez Roux, Daniel A. Rodríguez, SALURBAL

**Affiliations:** a Urban Health Collaborative, Drexel Dornsife School of Public Health, Philadelphia, USA; b Department of Environmental and Occupational Health, Drexel Dornsife School of Public Health, Philadelphia, USA; c Department of Epidemiology and Biostatistics, Drexel Dornsife School of Public Health, Philadelphia, USA; d National Centre for Medium Range Weather Forecasting, Ministry of Earth Sciences, Government of India, India; e Institute for the Environment, University of North Carolina at Chapel Hill, Chapel Hill, USA; f Department of Preventive Medicine, University of Sao Paulo Medical School, Sao Paulo, Brazil; g Department of Environmental Science, Policy & Management, University of California, Berkeley, USA; h Department of Landscape Architecture & Environmental Planning, University of California, Berkeley, USA; i Observatório de Saúde Urbana de Belo Horizonte, Universidade Federal de Minas Gerais, Belo Horizonte, Brazil; j Department of City and Regional Planning and Institute of Transportation Studies, University of California, Berkeley, USA

**Keywords:** Climate change, Demographic changes, Population aging, Latin America, Temperature-related mortality

## Abstract

**Background:**

In Latin America, climate change, urbanization, and an aging population are intensifying health risks from extreme temperatures. To accurately assess future temperature-related mortality impacts, evidence that integrates key demographic factors—such as the dynamics of population age composition, mortality rates, and population size—is essential.

**Methods:**

We projected the impact of nonoptimal temperatures on all-age and age-specific mortality during 2045–2054 for 326 cities in Latin America. Our analysis combined city-level daily mortality counts, gridded temperature data, downscaled and bias-corrected temperature simulations, and demographic data. We projected temperature-mortality impacts under two climate change scenarios while also considering changing population size, age structure, and age-specific mortality rates.

**Findings:**

By 2045–2054, the percentage of heat-attributable deaths under the most extreme temperature scenario will more than double from 0.87 % (95 % CI 0.77, 0.96) to 2.06 % (95% CI 1.80, 2.33), but cold-related mortality will decrease. Population growth and aging will exacerbate heat-related risks and offset reductions in cold-related deaths. For example, changes in population age structure will drive an increase in the heat-related mortality rate of 176% from baseline for a moderate temperature scenario.

**Interpretation:**

Beyond temperature changes, demographic shifts—particularly population growth and aging—will significantly amplify mid-century temperature impacts on mortality, underscoring the need for targeted climate adaptation and public health strategies.

## 1. Introduction

In Latin America, the convergence of climate change, urbanization, and an aging population has made extreme ambient temperatures a critical public health concern for the 21st century (Kephart et al., 2022). Latin America is projected to experience substantial increases in extreme temperatures from climatic changes due to anthropogenic greenhouse gas emissions (Intergovernmental Panel on Climate Change, 2023; Hartinger et al., 2023) which are increasing the frequency, intensity, and geographic extent of human exposure to extreme temperatures (Ramarao et al., 2024; Feron et al., 2019). Latin America is also highly urbanized, with 80 % of residents living in urban areas (United Nations Department of Economic and Social Affairs Population Division, 2019) prone to elevated risk of exposure to extreme heat (Ju et al., 2023) due to the urban heat island effect (Tuholske et al., 2021; Zhao et al., 2014). Furthermore, the population of Latin America is aging faster than the global average due to a combination of rising life expectancy, falling mortality rates, and decreasing birth rates (United Nations Department of Economic and Social Affairs Population Division, 2019). From 2020 to 2050, the proportion of the population aged 65 years or more is projected to more than double, from 9 % to 19 % (United Nations DoEaSA, 2022).

Older adults are particularly susceptible to health risks from extreme temperatures due to age-specific physiological factors, including a reduced ability to regulate body temperature and the presence of pre-existing chronic diseases, which inhibit the body’s ability to respond to extreme temperature exposures (Meade et al., 2020; Ebi et al., 2021). Existing projections of the health impacts of extreme temperatures under climate change scenarios estimate alarming increases in heat-related mortality (Gasparrini et al., 2017), yet these may be underestimated if changes in population composition are not accounted for in settings with growing populations of older adults (Cole et al., 2023; Chen et al., 2020; Yang et al., 2021). Population aging is likely to be a major driver of future health impacts of climate change-induced extreme temperatures by expanding the number and proportion of the population that is more sensitive to adverse health effects of extreme temperatures (Cole et al., 2023; Chen et al., 2020; Yang et al., 2021). In turn, the health risks from cold temperatures are also elevated among older adults (Kephart et al., 2022) and may be reduced or exacerbated by climate change in some geographic regions, yet this remains less well studied.

Other demographic and health factors may also influence the burden of temperature-related mortality. In recent decades, Latin America has experienced falling mortality rates (i.e. public health improvements) that are projected to continue to improve in the coming decades (United Nations DoEaSA, 2022). However, it is not known whether and to what extent these public health improvements might offset increasing population vulnerability to extreme temperatures due to population aging (Chen et al., 2024). Population projections indicate that the number of people living in Latin America and the Caribbean will increase from roughly 650 million as of 2023 to a projected 750 million in 2050 (United Nations DoEaSA, 2022). Thus, even a relatively small increase in the risk of death from nonoptimal temperatures may result in a significant increase in the number of temperature-related fatalities. Evidence that incorporates multiple demographic changes, including population age composition, age-specific mortality rates, and population size is needed for a nuanced understanding of the future burden of temperature-related mortality and to inform urban planning and policies for climate adaptation that protect the public’s health now and in the future.

To improve our understanding of the anticipated burden of extreme temperatures on health in the mid-21st century and to inform climate change mitigation and adaptation efforts to prevent temperature-related deaths in Latin America in the coming decades, we projected the impact of nonoptimal temperatures on all-age and age-specific mortality during the period of 2045–2054. Our analysis considered two climate change scenarios as well as changes in population size, age structure, and age-specific mortality rates across 326 cities in nine Latin American countries. We also decomposed the individual contributions of changes in temperature, population size, age-specific mortality rates, and population age structure to the projected temperature-related mortality to understand the importance of these factors for mortality impacts.

## 2. Methods

### 2.1. Study setting

This study was part of the Salud Urbana en América Latina (SALURBAL) project and conducted in accordance with the SALURBAL study protocol approved by the Drexel University Institutional Review Board (ID no. 1612005035). The SALURBAL project has gathered and standardized data on environmental, social, and health characteristics for all cities of ≥ 100,000 residents (N = 371 cities) in 11 Latin American countries (Diez Roux et al., 2019). These cities, ranging from small to megacities, were defined as urban agglomerations containing > 100,000 residents as of 2010 (Quistberg et al., 2019) and are composed of clusters of administrative units encompassing the urban built-up area as identified using satellite imagery (Quistberg et al., 2019). Our analysis includes 326 cities in Argentina, Brazil, Chile, Costa Rica, El Salvador, Guatemala, Mexico, Panama and Peru. Cities in Colombia and Nicaragua were excluded due to the limited availability of daily mortality data.

### 2.2. Baseline temperature

We estimated the population-weighted daily mean ambient temperature for each city from 2002 to 2015 using the ERA5-Land climate reanalysis with native ~9-km horizontal resolution (Muñoz-Sabater et al., 2021). Additional details have been previously published (Kephart et al., 2022) We used estimates of air temperature at 2 m above the land surface, which we refer to as ambient temperature, as a proxy for health-relevant environmental exposure. We calculated daily mean temperatures by averaging ERA5-Land hourly temperatures by calendar days. To better approximate population exposures, we spatially weighted city temperature using 2010 estimates of the spatial distribution of the population (WorldPop, https://www.worldpop.org: Argentina, Brazil, Chile, Costa Rica, El Salvador, Guatemala and Mexico) or urban footprint (Global Urban Footprint, https://www.un-spider.org/node/11424: Panama and Peru).

### 2.3. Temperature projections

We projected temperatures for the 2045–2054 period under Representative Concentration Pathways 2.6 (RCP2.6) and 8.5 (RCP8.5), the upper and lower bound future scenarios approved by the Intergovernmental Panel on Climate Change (IPCC) (Intergovernmental Panel on Climate Change, 2023). RCP2.6 and RCP8.5 represent greenhouse gas emissions under scenarios of immediate dramatic global reductions in emissions and business-as-usual emissions, respectively. We chose these two scenarios to represent an upper and lower boundary of plausible climate scenarios and to understand the range of plausible impacts on temperature-related mortality in the mid-21st century. We conducted dynamic downscaling of historical and two future temperature projections using a single Earth System Model (ESM). We used the low-resolution version of the Max-Planck-Institute Earth System Model (Giorgetta et al., 2013) (MPI-ESM-LR; referred to as MPI hereafter) from the Coupled Model Intercomparison Project (CMIP5) (CMIP6 was not available when we started the simulations) to downscale to a 12 km × 12 km horizontal resolution over Latin America using the Weather Research Forecast (WRF) model v4.1.4 (Skamarock et al., 2019). The WRF model was configured for two individual domains over Latin America: Central America and Mexico (CAM) and South America (SAM). We performed three downscaled simulations for each domain, one for the historical period (1996–2005) and two future simulations (2045–2054) under RCP2.6 and RCP8.5 emissions scenarios. See the online appendix of Ramarao et al. (Ramarao et al., 2024) for more technical details regarding the downscaling. We applied a statistical bias correction introducing a constant offset factor to adjust for differences between simulated and observed monthly mean temperatures in the historical period (Hempel et al., 2013) at each grid point. As explained in Ramarao et al. (Ramarao et al., 2024), we used a trend-preserving bias correction method developed by Hempel et al (Hempel et al., 2013). In addition to correcting bias in the monthly mean temperature (offset), this method also modifies the daily variability of the simulated temperature data about their monthly means to match the observed daily variability in the historical period (1996–2005). This method was developed within the first Inter-Sectoral Impact Model Intercomparison Project (ISI-MIP) (Warszawski et al., 2014; Sillmann et al., 2013) to project the impacts of climate change across multiple sectors including agriculture, water, biome, health, etc. We used hourly temperature data at a 9 km × 9 km horizontal resolution from ERA5-Land as the reference dataset. The same offset factors were then applied to model simulations of the future, mid-century period (2045–2054). Model outputs were bilinearly interpolated to ERA5-Land grids before applying bias correction. Additional details on the resulting methodology and dataset have been previously published (Ramarao et al., 2024).

We applied projected increases in daily temperature from the historical period to the mid-century period to the baseline ERA5-Land observations using the mean temperature for each calendar day over the 10-year projection period (2045–2054). The final output was a dataset of projected temperature for each day over the course of the 10-year period under each of the two emissions scenarios (RCP2.6 and RCP8.5) for each city.

### 2.4. Population projections

We used the United Nations (UN) World Population Prospects database to obtain country-level demographic data (United Nations DoEaSA, 2022). We aggregated the UN estimates of each country’s total population in 5-year age groups to population counts for broader age groups a=0−49, 50–64, and 65+ and the proportion, $\pi^{a}$, of each country’s population in these age groups for the baseline periods (specific for each country) and 2045–2055. These age groups were needed for consistency with our prior analyses of temperature-mortality associations (Kephart et al., 2022; Bakhtsiyarava et al., 2023; Schinasi et al., 2023). We also used the abridged life tables from the UN World Population Prospects database (United Nations DoEaSA, 2022) (both sexes combined; 5-year age increments) to derive projected country-level mortality rates at mid-century (2045–2054) and baseline (2002–2015) under a “medium” projection scenario (i.e., assuming medium fertility, mortality, and international migration (United Nations DoEaSA, 2022). The life tables describe the mortality rates applicable to a group of individuals born in the same year as they progress from age 0 to 100. We used the probability of dying (hereafter referred to as mortality rate) from the life tables for 5-year age increments to compute average mortality rates, $r^{a}$, for the three age groups of interest in our study (a=0−49, 50–64, and 65+) for the baseline and mid-century periods. As described below these age-specific annual mortality rates are then used to estimate daily deaths by age group for the baseline and mid-century periods.

### 2.5. Daily mortality data

We compiled baseline individual-level mortality data and population data from vital registration systems in each country (Bilal et al., 2021). Mortality and population data for Brazil, Chile, and Mexico were downloaded from publicly available repositories of statistical agencies in each country. Data for Argentina, Costa Rica, El Salvador, Guatemala, Panama, and Peru were obtained directly from each country’s respective statistical agency. Individual mortality records included age and date of death. We aggregated individual death records into daily counts of deaths by age group for each city. Within each city, the observed mortality counts on day j for each age group a are denoted as $D_{j}^{a}$. Note that, although these counts are observed in the baseline period, they can be expressed as $D_{j}^{a}=N^{*}\pi^{a*}r^{a*}s_{j}^{a}$, where N is the total city population; $\pi^{a}$ is the proportion of age group a in the city; $r^{a}$ is the average annual mortality rate for age group a (deaths in the age group divided by population in the age group); $s_{j}^{a}$ is the observed proportion of all deaths in age group a over the year that occur on calendar day j (used to capture seasonal variations).

### 2.6. Projected daily mortality considering demographic changes

The projected number of total deaths on day j in in the mid-century can be written similarly as $D_{jf}^{a}=N_{f}*\pi_{f}^{a}*r_{f}^{a}*s_{jf}^{a}$, but instead using the projected total population counts in the future time period, $N_{f}$; the share of the age group a in the total population in the future, $\pi_{f}^{a}$; and projected mid-century mortality rate, $r_{f}^{a}$, where the quantities $N_{f},\pi_{f}^{a},r_{f}^{a}$, were approximated from the UN demographic tables described above. Since future seasonality patterns are not available, we assumed that the daily seasonal risk of death $s_{\text{jf}}^{a}$ is the same as observed in the baseline period $s_{\text{jf}}^{a}=s_{j}^{a}$. Hence, the total number of future deaths for age group a on day j can be expressed as

(1) $$D_{\text{jf}}^{a}=D_{j}^{a*}\frac{N_{f}}{N}*\frac{\pi_{f}^{a}}{\pi^{a}}*\frac{r_{f}^{a}}{r^{a}}=D_{j}^{a*}w_{a}$$

where $w_{a}$ represents age-group-specific weights reflecting the combined differences in daily mortality associated with changes in total population size, mortality rates, and age composition between the baseline and mid-century period.

We constructed four mortality series considering various changes in the population. Using Eq. (1), for the first mortality series including all three considered demographic components (population counts, age-specific mortality rates, and population age structure), the weights were constructed using country-level baseline and future total population counts, age-specific mortality rates, and population age structure. Specifically, we used baseline and future country-specific probabilities of dying for every age group from the UN life tables and projected population counts to derive the components of the weights. The supplementary second, third, and fourth mortality series included changes in temperature and one of the following: population size, age-specific mortality rates, or population age structure, to demonstrate the independent contribution of these various factors to the change in temperature-related mortality.

To estimate future daily mortality rates for each city, we computed age-specific city-level counts of daily average observed mortality for every calendar day (average mortality for January 1, January 2, etc.) in the mid-century scenario ten-year period. This allowed us to preserve the seasonal pattern of age-specific mortality for each city.

Thus, we generated four projected mortality series: (1) using projected values for all components of the weights; (2) using ratio Nf/N, but keeping all other ratios = 1; (3) using the ratio $\pi_{f}^{a}/\pi^{a}$, but keeping all other ratios = 1; (4) using ratio $r_{f}^{a}/r^{a}$, but keeping all other ratios = 1. Next we describe how we used these mortality series to project the impact of future temperature on mortality under two temperature scenarios.

### 2.7. Quantification of mortality impacts

We used established methods described by Gasparrini et al. (Gasparrini et al., 2017) and Vicedo-Cabrera et al. (Vicedo-Cabrera et al., 2019) to obtain the projected number of deaths attributable to non-optimal temperature. Briefly, these methods rely on (1) the estimation of temperature-mortality associations using the baseline mortality and temperature data, (2) projecting temperature-mortality associations using the mid-century temperature data and projected demographic changes, and (3) computing the projected number of temperature-related deaths which are then subsequently used to compute excess death fractions and temperature-related mortality rates.

We projected temperature impacts on mortality using two types of metrics and using combinations of the two climate scenarios and four scenarios for population changes for a total of eight scenarios. The outcome metrics are the excess death fractions (EDFs), defined as the number of deaths that are attributable to non-optimal temperature divided by the total number of deaths (Gasparrini and Leone, 2014); and temperature-related mortality rates (TMRs), defined as number of deaths attributable to non-optimal temperature per 1,000 population. While the EDFs demonstrate the proportion of a population affected by temperature-related mortality, the TMRs describe the frequency of temperature-related mortality impacts and enable standardized comparisons of temperature impacts across different population sizes.

The derivation of the measures of mortality impacts described above – EDFs and TMRs – relies on estimating the projected number of deaths due to non-optimal temperatures for each age group a on a given day j, using:

(2) $$D_{\text{jf},\;\text{temperature}}^{a}=D_{jf}^{a}*\left(1-e^{-\left(f_{a}\left(T_{\text{proj},j}\right)-f_{a}\left(T_{\text{mm}}\right)\right)}\right)$$

These attributable deaths are a function of the total number of deaths ($D_{\text{jf}}^{a}$) on day j, age- and city-specific temperature-mortality association curves $f_{a}$, projected ambient temperatures on day $j\left(T_{\text{proj},j}\right)$, and the city- and age-specific minimum mortality temperature ($T_{\text{mm}}$). Note that the projected temperature-related deaths are dependent on population changes because the total number of deaths ($D_{\text{jf}}^{a}$) depends on the population change scenario as described above. We used the city- and age-specific temperature-mortality curves, $f_{a}$, from our previously published analysis of ambient temperature and mortality in the same 326 Latin American cities (Kephart et al., 2022). These age-specific temperature-mortality curves were derived using distributed lag nonlinear models with distributed lags of 0–21 days and estimated for each of the three age groups: 0–49, 50–64, and 65+ years. Because climate change is expected to increase the range of ambient temperatures at a given location beyond the observed historical temperatures, we performed a log-linear extrapolation of the baseline city-specific temperature-mortality curves to the lower and upper bounds of projected temperatures (Vicedo-Cabrera et al., 2019). The projected age-specific temperature-related deaths $D_{\text{jf},\text{temperature}}^{a}$ were calculated for each of 8 scenarios (2 temperature scenarios x 4 population scenarios), for each day in each city, and subsequently aggregated to produce the specific metrics, as follows.

We calculated age-group-specific counts of deaths attributable to heat only, cold only, and heat and cold combined by summing $D_{\text{jf},\text{temperature}}^{a}$ within specific temperature ranges ($T_{\text{proj},j}<T_{\text{mm}};T_{\text{proj},j}>T_{\text{mm}}$; and all temperatures). For each temperature range, we then computed two metrics: excess death fractions (EDFs) and temperature attributable mortality rates (TMRs). We estimated EDFs for heat and cold by dividing the number of deaths attributed to heat and cold, respectively, by the number of total projected deaths in the population, presented as a percentage. The age-group-specific numbers of deaths attributable to temperature (heat only, cold only, and heat and cold) were summed up to obtain the total (across all ages) absolute number of temperature-related deaths (in each temperature range) and total (across all ages) EDFs. The reported point estimates and confidence intervals were obtained by running 1,000 Monte Carlo simulations for every city for every scenario, to account for estimation of the temperature-mortality association curves, $f_{a}$. Point estimates represent the average coefficient value across the simulations, and the empirical confidence intervals (eCI) represent the 2.5th and 97.5th percentiles of the distribution of the simulated coefficients. To obtain region-wide heat and cold EDFs across all study cities, we added city-level heat- and cold-related deaths from each simulation and divided them by the sum of city-level deaths from each simulation across all cities. The region-wide point estimates and confidence intervals were obtained as the average coefficient value across all simulations and the 2.5th and 97.5th percentiles of the distribution of the simulated coefficients.

Additionally, we estimated heat- and cold-related mortality rates by dividing the number of deaths in each age group attributed to heat and cold, respectively, by the total city population in each age group. For the baseline period, the rates were computed using baseline city population in each age group. For the future period, city population in each age group was projected by multiplying the baseline city population by the country-level population growth factor (ratio of the future to baseline population) obtained from the UN projections. Point estimates, confidence intervals, and region-wide estimates were obtained using 1,000 Monte-Carlo simulations for each city for every scenario, analogous to those for EDFs described above.

### 2.8. Contribution of climate change and demographic changes

Finally, we decomposed the individual contributions of changes in temperature, population size, age-specific mortality rates, and population age structure to projected temperature-related mortality. To estimate the impacts of temperature changes alone, we projected EDFs and heat- and cold-mortality rates using mid-century temperature projections applied to baseline demographics. To estimate the impact of population size changes alone, we estimated mortality outcomes under scenarios with temperature and population size changes, then subtracted the mortality impact of temperature changes. We repeated similar analyses but replacing changes in population size with changes in population mortality rates or population age structure, independently.

## 3. Results

### 3.1. City temperature projections

**Fig. 1.**
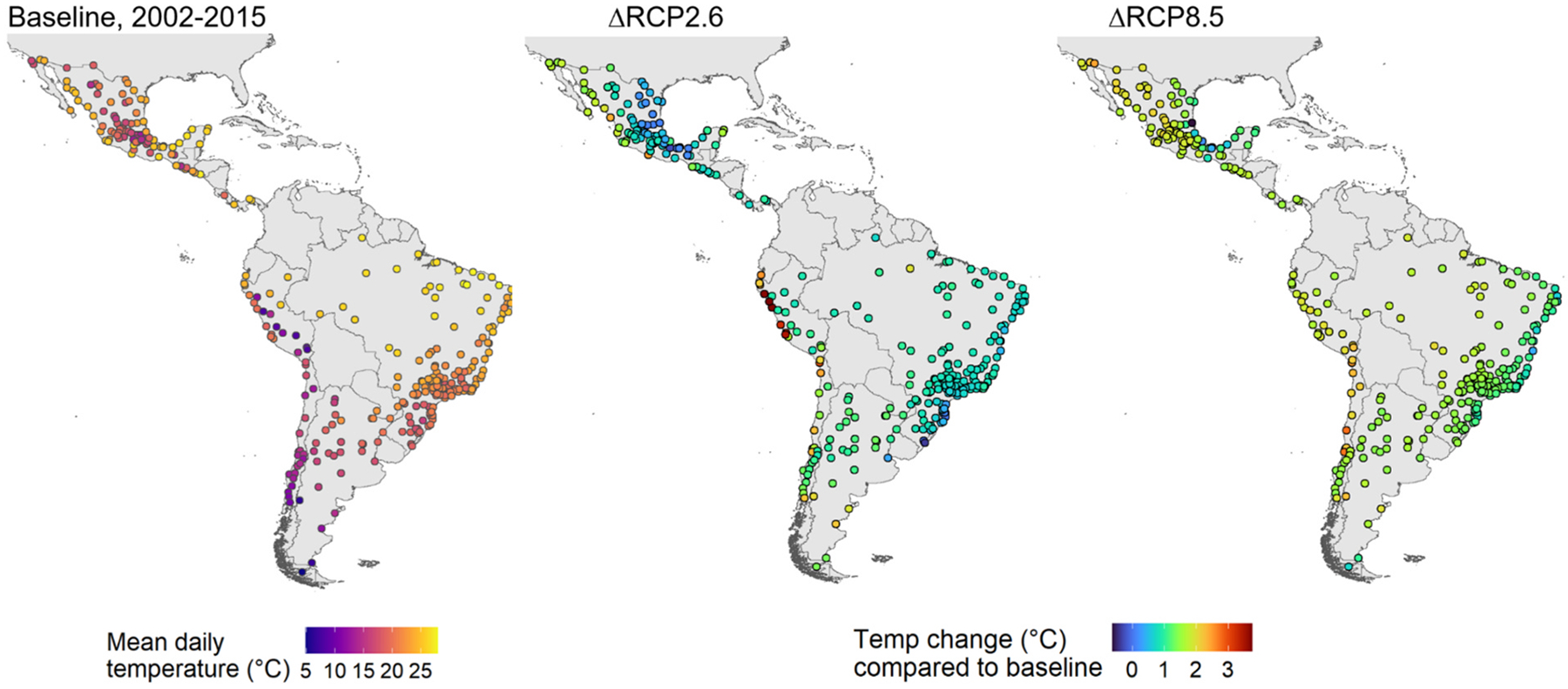
City-level daily mean temperature at baseline period and projected changes in daily mean temperature from baseline period to 2045–2054 under RCP2.6 and RCP8.5 greenhouse gas emissions scenarios.

In Table 1, we present baseline population, age distribution, deaths, and mean temperatures for the 326 study cities, overall and by country. The median city-specific percentage of the population aged 65 years or older was 6.7 % overall, ranging from 5.5 % in cities in Peru to 8.8 % in cities in Chile. In Fig. 1 and Supplemental Table S1, we present baseline temperatures and changes in the ten-year average mean daily temperatures under RCP2.6 and RCP8.5 scenarios for each city, relative to the baseline period. As expected, we found wide variation in expected increases in temperature by city and region. Most cities are projected to experience an increase in mean daily temperature under both emission scenarios. For RCP2.6, mean daily temperature across all the cities is projected to rise by 1 °C (IQR 0.8, 1.2), while under RCP8.5 the projected increase is 1.7 °C (IQR 1.4, 2.0). Under RCP2.6, the strongest warming trends in the region were projected along the Pacific coast of Peru. Temperature increases under RCP8.5 are higher overall than under RCP2.6 and increases are broadly distributed throughout cities in Chile, Peru, Argentina, and Brazil as well as the majority of Central America and Mexico.

### 3.2. Country demographic projections

In Supplemental Table S2, we present projected changes to population size, age-specific mortality rates, and population age structure, overall and by country. From baseline to 2045–2054, all nine countries will experience population growth, and their total population is projected to increase by 25 %. There is also a trend of decreasing age-specific death rates (i.e. a healthier population) across all age groups and countries. Finally, there are decreases in the shares of younger populations and increases in population aged 50+ years. Across the nine study countries, the percentage of the population aged 65+ years is projected to increase from 7 to 19 % by the mid-21st century. Taken together these trends suggest overall population growth and improvements in life expectancy reflected by declining death rates across age groups and growing proportions of the population who are older adults.

### 3.3. City-specific mortality projections under temperature scenarios

**Fig. 2.**
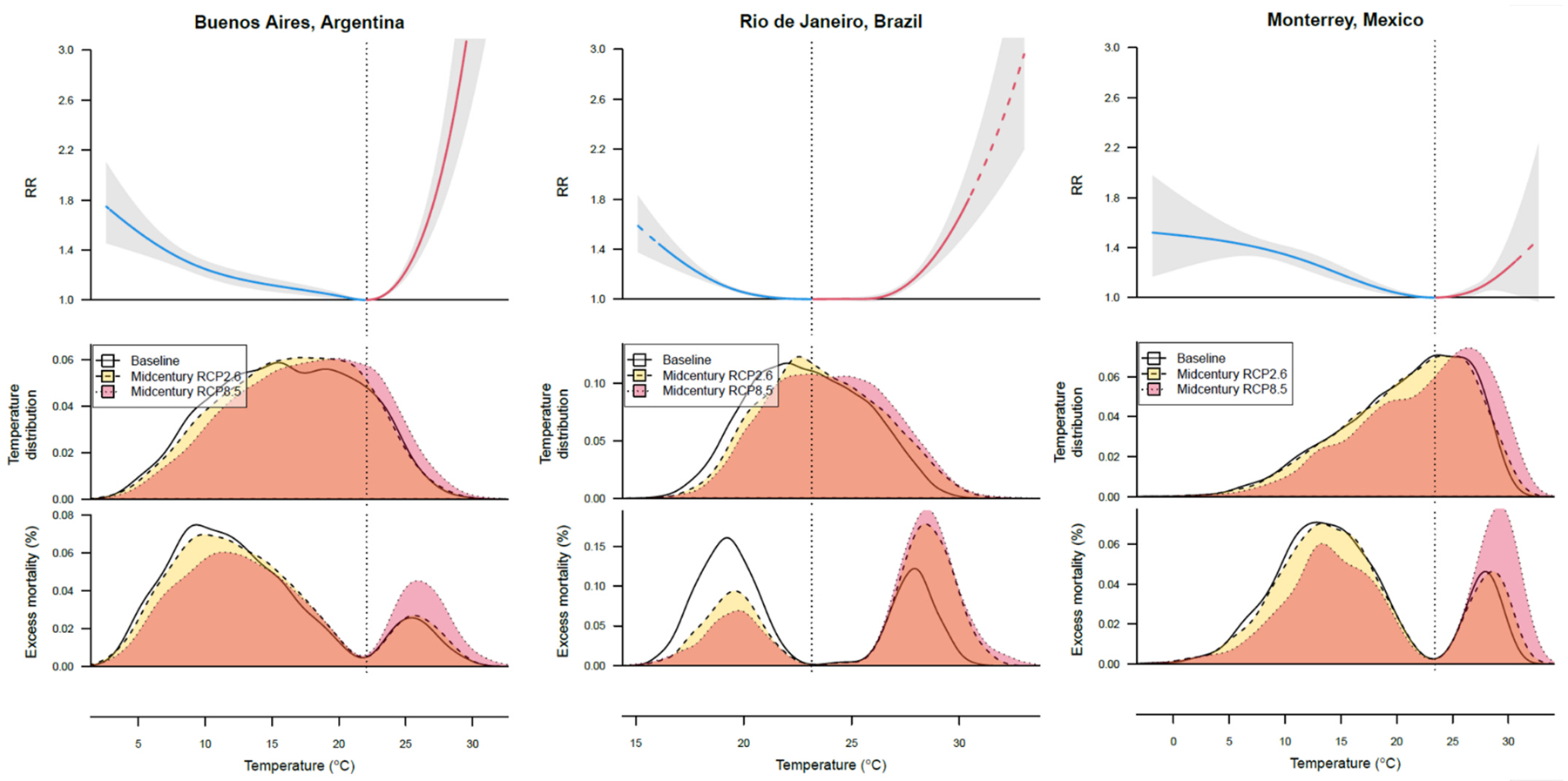
Present and mid-century temperature-related mortality in three selected Latin American cities. The top panel depicts a relative risk (RR) of mortality across the range of mean daily temperatures with 95% empirical confidence interval shaded in gray. The dotted vertical line is the minimum mortality temperature (MMT), or the temperature at which the temperature-mortality risk is the lowest. MMT splits the temperature-mortality curve into cold-related mortality (blue) and heat-related mortality (red). The middle panel shows the distribution of mean daily temperatures for the baseline period and for the mid-century period in accordance with RCP2.6 and RCP8.5. The lower panel shows the distribution of excess death fractions associated with baseline temperature and the two mid-century scenarios (RCP2.6 and RCP8.5), given the city-specific temperature-mortality risk curve (top panel) and observed and mid-century projected temperatures (middle panel). The projections in the figure account for changes in temperature only and do not account for changes in population size, age-specific mortality rates, and population age structure.

As an example for three cities, Fig. 2 depicts city-specific relative risk (RR) of mortality by temperature, the distribution of daily temperatures under baseline, RCP2.6 and RCP8.5 scenarios, and excess mortality from hot and cold temperatures in the baseline and mid-century periods, assuming baseline population size, age-specific mortality rates, and age distribution.

As expected, the mortality impacts of temperature at baseline and under mid-century temperature projections vary by city. Under RCP8.5, Buenos Aires is projected to experience a decrease in cold-related excess death fractions, from 11.58 % (95 % CI 9.45, 13.74) to 9.54 % (95 % CI 7.62, 11.53) of all deaths. However, the share of deaths attributable to heat (heat EDF) is projected to increase by + 1.36 percentage points (95 % CI 0.69, 2.03) due to an increase in the frequency of hot days. Rio de Janeiro is projected to experience extreme hot temperatures beyond the range of maximum temperatures observed at baseline. Two factors − high mortality risk under extreme hot temperatures and a projected increase in the fraction of days in a year with hot temperatures contribute to an increase in heat EDF from 1.63 % of all deaths at baseline to 3.58 % of all deaths at mid-century, for a net increase of + 1.95 percentage points (95 % CI 1.36, 2.53). The cold EDF in Rio de Janeiro is estimated to decrease by 1.10 percentage points (95 % CI −1.70, −0.51). Monterrey is projected to experience changes in temperature-related excess mortality similar to those in Buenos Aires and Rio de Janeiro, although the heat-mortality risk curve is not as steep as in the other two cities. Heat EDF is projected to increase by + 1.88 percentage points (95 % CI −0.69, 4.45) and cold EDF is projected to decrease by 1.6 percentage points (95 % CI −2.78, −0.42), for a total increase of + 0.28 % percentage points (95 % CI −2.69, 3.25) in the temperature-related excess mortality by mid-century.

### 3.4. City-level mortality projections under temperature and demographic scenarios

**Fig. 3.**
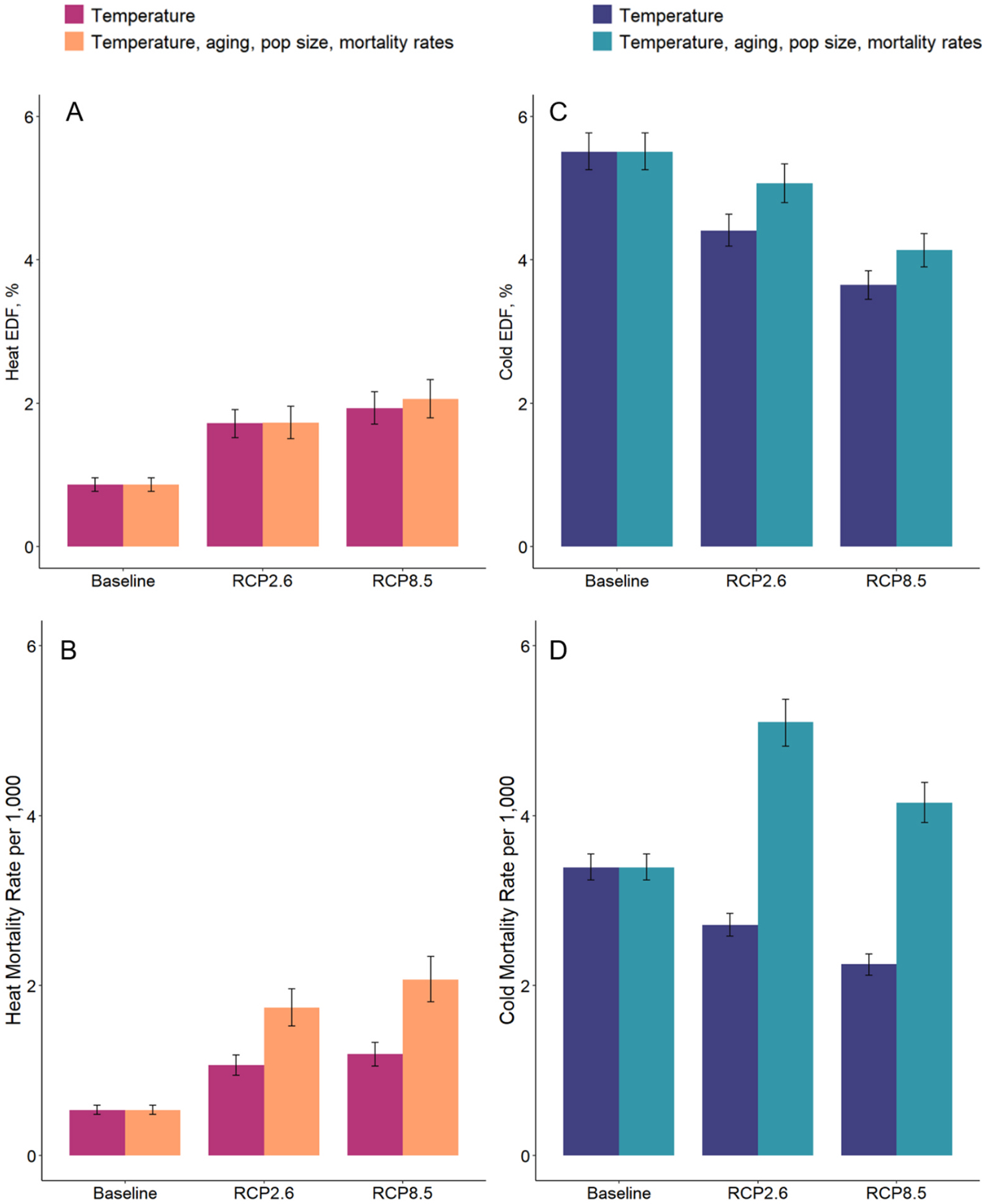
Heat and cold excess death fractions and temperature-related mortality rates for all 326 Latin American cities at baseline (2002–2015) and mid-century (2045–2054) periods under two scenarios of temperature changes (RCP2.6 and RCP8.5) with and without expected changes in the population size, age-specific mortality rates, and population age structure at mid-century. Panels A and B relate to excess death fractions (EDF)^1^ from heat and cold; panels C and D relate to heat- and cold-related mortality rates^2^. ^1^Excess death fractions represent the percentages of deaths attributable to temperature (e.g., heat, cold) among all deaths in a population. ^2^Temperature-related mortality rates represent the number of deaths attributable to temperature (e.g., heat, cold) per 1,000 population.

Fig. 3 shows the heat- and cold-related mortality across the entire sample of 326 cities for the baseline period, as well as the two mid-century temperature scenarios with and without mid-century demographic changes for the two outcomes. Across all 326 cities, excess deaths associated with heat are projected to increase under both emissions scenarios, but a greater increase is expected under RCP8.5 (+1.20 percentage points of all deaths compared to baseline, 95 % CI 0.92, 1.48) than under RCP2.6 (+0.87 % percentage points of all deaths compared to baseline, 95 % CI 0.63, 1.11) (Table 2). The cold EDF is projected to decrease by 0.44 percentage points (95 % CI −0.81, −0.07) and 1.38 % percentage points (95 % CI −1.72, −1.03) for RCP2.6 and RCP8.5, respectively.

The direction of the results for the heat- and cold-related mortality rates are consistent with EDFs, except for cold-related mortality rates for the RCP2.6 and RCP8.5 scenarios with demographic changes. For example, the cold-related mortality rate is projected to change from 3.39 (95 % CI 3.24, 3.55) deaths per 1,000 at baseline to 5.10 deaths per 1,000 in RCP2.6 (95 % CI 4.82, 5.37). Therefore, even though warming temperatures in RCP2.6 and RCP8.5 will drive a decrease in the percentage of total deaths that occurred on days with cold temperatures, the total number of cold-associated deaths will increase due to a growing temperature-sensitive population. City-specific results are presented in Supplemental Figs. S1–S4 and country-specific results are presented in Supplemental Figs. S5–S8.

**Fig. 4.**
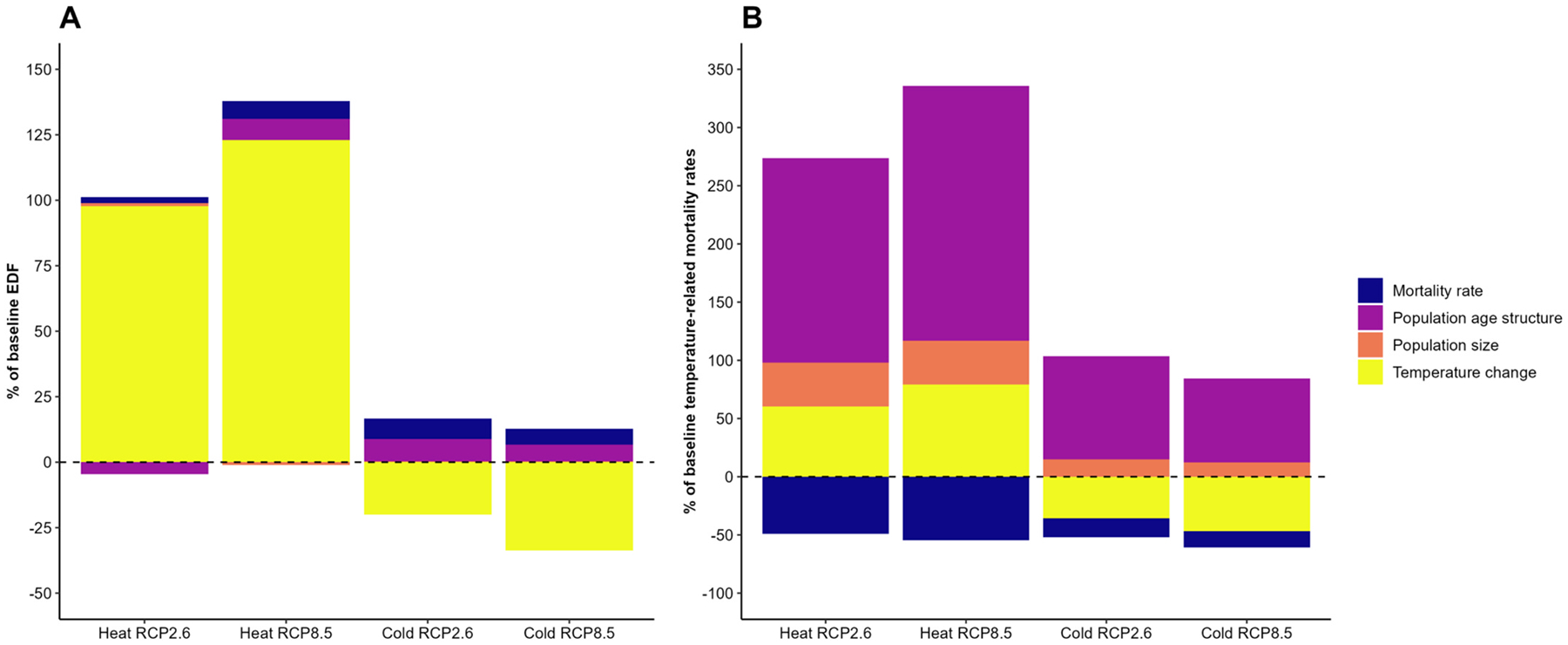
Contributions of changes in temperature, population size, age-specific mortality rates, and population age structure to projected changes in heat- and cold-EDFs^1^ (panel A) and heat- and cold-related mortality rates^2^ (panel B) from baseline to mid-century. Changes in EDFs and temperature-related mortality rates are expressed as percentage changes of their corresponding baseline values. X axis for panel A: Heat RCP2.6 and Heat RCP8.5 are the heat EDF under RCP2.6 and RCP8.5, Cold RCP2.6 and Cold RCP8.5 are cold EDFs under RCP2.6 and RCP8.5. X axis for panel B: Heat RCP2.6 and Heat RCP8.5 are heat-related mortality rates under RCP2.6 and Heat RCP8.5, Cold RCP2.6 and Cold RCP8.5 are cold-related mortality rates under RCP2.6 and RCP8.5. ^1^ Excess death fractions represent the percentages of deaths attributable to temperature (e.g., heat, cold) among all deaths in a population. ^2^ Temperature-related mortality rates represent the number of deaths attributable to temperature (e.g., heat, cold) per 1,000 population.

Changes in the population size, age-specific population mortality rates, and population age structure will impact heat- and cold-related mortality in different directions (Fig. 4). For EDFs, the contributions of these demographic changes to excess mortality are relatively small compared to the much larger impacts of temperature changes from climate change. As presented in Fig. 4A, without projecting any changes to the population age structure, population size, or mortality rates, heat EDF under RCP2.6 will increase by 98 % from baseline. Changes in the population age structure will decrease heat EDF by 5 % relative to its baseline value, while changes in mortality rates and population size will result in small heat EDF increases of a few percent from baseline. Under RCP8.5, heat EDF will grow by 123 % from baseline due to increases in temperature alone. Changes in population mortality rates and population age structure will also exacerbate heat EDFs under RCP8.5. Cold EDF is projected to decrease by 20 % and 34 % from its baseline value under RCP2.6 and RCP8.5, respectively, due to changes in the temperature alone. Nevertheless, changes in the population mortality rate and population age structure will somewhat offset the reduction in cold-related excess deaths from climate change in both RCP scenarios.

The contribution of demographic factors is much greater (and inversely, the contribution of temperature changes is smaller) for temperature-related mortality rates compared to EDFs, where temperature is the dominant driver. As Fig. 4B shows, temperature changes will contribute to an increase in heat-related mortality rate of between 60 % and 80 % (depending on the emissions scenario) from baseline. Changes in the population age structure and population size will amplify heat-related mortality rates for both scenarios: population age structure will drive an increase in heat-related mortality rate of 176 % and 219 % from baseline for RCP2.6 and RCP8.5, respectively, while changes in the population size will increase the baseline heat-mortality rates by about 38 % in each scenario. Warming temperatures will contribute to a decrease of 36 % and 47 % in cold-related mortality rates from baseline for RCP2.6 and RCP8.5, respectively. However, changes in the population size and age structure will offset reductions in cold-related mortality rates in both scenarios. Finally, expected changes in mortality rates (i.e. improving public health) will decrease heat- and cold-related mortality rates for both scenarios, but these decreases will not be sufficient to offset the increases in temperature-mortality rates driven by climate change and other demographic changes.

## 4. Discussion

In 326 Latin American cities, we projected the mid-21st century burden of cold- and heat-related mortality under RCP2.6 and RCP8.5 climate scenarios while accounting for future demographic changes in population size, age-specific population mortality rates, and population age structure. From baseline (2002–2015) to mid-century (2045–2054), our analysis projects a doubling of the percentage of total deaths attributable (excess death fraction, EDF) to heat under RCP2.6 and a 2.4-fold increase under RCP8.5. In the same period, cold EDF will decrease (due to warming temperatures), by 8–25 %. Heat-related deaths per 1,000 population (temperature-mortality rate, TMR) will similarly increase under both emissions scenarios, with a 3.3-fold increase under RCP2.6 and a 3.9-fold increase under RCP8.5. Despite warming temperatures, cold-related deaths per population (TMR) will grow by 22–50 %, driven by increasing population vulnerability due to aging.

Rising temperatures under both climate scenarios are expected to increase heat-related deaths and decrease cold-related deaths. However, growing population size and aging populations will act to both amplify heat risks and limit or overwhelm corresponding decreases in cold-related mortality. These demographic changes are major drivers that will compound the impact of projected temperature changes on deaths in the region. When examining the relative measure of EDFs, the primary driver of changes in heat- and cold excess mortality are changing temperatures due to climate change from greenhouse gas emissions, and the contributions of demographic changes to the overall excess mortality burden are comparatively small.

Population aging and (falling) mortality rates will contribute to increases in heat and cold EDFs across most of the scenarios. One exception is a decrease in heat EDFs under RCP2.6 associated with population aging. A combination of the declining risk of heat-mortality among those 50–64 (Supplemental Table 3) and their growing share in the population (Supplemental Table 2) pulls down the overall heat EDF under modest warming observed under RCP2.6. Also, because the temperature–mortality relationship is nonlinear, small increases in temperature (like what we see under RCP2.6) may not lead to proportionally large increases in heat-related deaths unless there are sharp increases in the relative risk of mortality such as those occurring along the steepest portions of the curve. Declining mortality rates will amplify heat and cold EDFs compared to the baseline. Even if the relative risk of temperature-related mortality declines due to a healthier population – resulting in fewer heat- or cold-related deaths (the numerator for the EDF) – the number of total deaths (the EDF denominator) may decline even more rapidly, also due to improved population health. This can lead to an increase in EDF. So, temperature-related deaths may comprise a larger share of all deaths not because they are increasing in absolute terms, but because other causes of death are declining more quickly. This trend does not necessarily indicate worsening health outcomes overall, but – because EDF is a relative metric – rather highlights the growing relative importance of temperature-related mortality within a population.

For RCP2.6, we also observe that the risk of heat-related mortality is projected to increase for those 0–49 and 65+ (Supplemental Table 3). The trend is similar for a hotter scenario, RCP8.5, except that for 50–64-year-olds the risk of mortality at extreme heat is projected to increase. Among the three age groups considered we observe the lowest baseline and projected heat-mortality risk for those 50–64. While few heat-mortality studies have focused specifically on 50–64 year olds, who can be considered a middle- to late-middle aged population, numerous studies have pointed out increased vulnerability to heat among the young and elderly (Benmarhnia et al., 2015). The 0–49 age group includes children, whose thermoregulatory systems are not fully developed, and who have a higher surface area-to-body mass ratio, resulting in reduced sweating efficiency and a slower ability to adapt to rising environmental temperatures (Vanos, 2015). The 0–49 group also includes adults of working age, many of whom may be employed in heat-exposed occupations like construction or agriculture (Habibi et al., 2024). The elderly’s (65+) vulnerability to heat is exacerbated because of impaired thermoregulation associated with aging, comorbidities, and social isolation (Meade et al., 2020; Vandentorren et al., 2006; Kenney and Munce, (1985) 2003). The 50–64 cohort may be less vulnerable to heat compared to the other age groups, particularly 65+, because they are healthier (e.g., fewer comorbidities than the 65+) and more economically secure. To the extent that mortality rates reflect the underlying population health, this is confirmed by the difference in age-specific mortality rates (Supplemental Table 2): the average mortality rate in the sample for 50–64 is almost 11 times lower than for those 65+ (0.043 per 1,000 compared to 0.467 per 1,000, respectively).

However, the absolute measures of deaths per population, heat- and cold-related temperature mortality rates (TMR) are strongly driven towards a greater mortality burden by population aging. While climate change will increase TMR from heat by 60 % to 80 % from baseline, changes in population age structure alone will increase TMR from heat by 176 % to 219 %. While public health gains in reducing underlying mortality rates will by definition also reduce deaths attributable to temperature, these reductions will be overwhelmed by increasing temperature exposures and increasing population vulnerability due to population aging. Given changes in temperatures alone, TMR from cold would decrease by almost 50 % from baseline under both climate scenarios. However, population aging will completely offset that decrease and lead to an increase of 72 % to 88 % in TMR from cold, depending on the emissions scenario.

Our findings from 326 Latin American cities are qualitatively similar to a study by Gasparrini et al. that included 32 locations in Brazil, Chile, and Mexico and found increasing heat mortality and decreasing cold mortality associated with increasing global warming from greenhouse gas emissions (Gasparrini et al., 2017). However, our findings are not easily comparable because our study and the Gasparrini study use different baseline and future time periods, which impacts the magnitude of expected temperature changes and the associated impacts on mortality. In 152 cities in Brazil, we estimated heat EDFs of 1.82 % and 2.43 % under RCP2.6 and RCP8.5 climate scenarios, respectively, for the period of 2045–2054. These EDFs are similar to Gasparrini et al. estimates for 18 cities in Brazil: 1.7 % and 3.3 % for the same climate scenarios during the marginally more distant period of 2050 to 2059. However, we estimated a higher burden of cold-related mortality in 2045–2054 than Gasparrini et al. The higher projections of cold-related mortality in our study may be due to our inclusion of a much larger, diverse sample of cities in the region, including a range of city sizes with as few as 100,000 residents where urban heat islands may have a limited buffering effect on population exposure to cold. Our findings that population aging will increase heat risk and limit reductions in cold risk in 326 Latin American cities is consistent with findings from Chen et al. from 800 locations globally (Chen et al., 2024). Chen and colleagues modeled future risk under scenarios of increases in global mean temperature and found that through the 21st century, population aging will increase the burden of heat-related mortality by 20–25 % and will substantially limit reductions in cold-related mortality from warming temperatures. This is consistent with our projections for the more immediate future (2045–2054) as presented in Fig. 3.

This study used state-of-the-art statistical methods to estimate temperature-mortality associations and quantify the projected temperature-mortality burden using downscaled and bias-corrected temperature data at a high resolution and population projections in a large sample of 326 cities in Latin America. A key strength and novel contribution of our study is the use of age-specific temperature-mortality responses to project expected impacts while accounting for demographic trends. In this study, we projected the future mortality count – and then expressed it as a future proportion of temperature-related mortality and future temperature-related mortality rates – while considering changing population characteristics that may impact the total number of deaths. These included population size, age-specific mortality rates, and population age composition. Our analysis also highlights that falling age-specific mortality rates (i.e. public health improvements) can decrease temperature-related mortality rates but that will not be enough to offset the increases in those rates due to climate change, population aging and overall population growth. A major strength and innovation of our study is the estimation not only of relative measures of temperature-associated deaths (EDFs) but also absolute measures of risk (mortality rates). These measures of absolute risks provide important information about the healthcare and population burdens of temperature and demographic changes on deaths per population, beyond the more commonly estimated relative proportions of deaths attributable to heat or cold. We found that age structure and population size changes have limited impacts on EDFs but have substantial impact on absolute rates of temperature-related deaths (TMRs). The analysis framework presented in this study provides a flexible way to account for shifting demographic phenomena when projecting temperature-related mortality burden.

In Latin America population aging is the largest demographic trend contributing to the increase in the total number of deaths across the population, and it is the largest demographic contributor to the changing population mortality burden from ambient temperatures. The projected absolute number of temperature-related deaths will grow not only because of general population growth but also because of an expanding population of older adults who are at higher risk of temperature-related mortality. According to the UN, 82 % of total deaths in Latin America in 2050 will occur among those ages 65+ (United Nations DoEaSA, 2022). Thus, significant efforts should be undertaken to protect the growing older population from the growing threat of extreme temperatures under climate change.

This study should be interpreted within the context of its limitations. We did not account for potential biological, technological, or other adaptations to the changing climate, and we assumed that seasonal patterns of mortality risk (within a calendar year) do not change in the future. However, there is currently no consensus on the best way to empirically account for adaptation in temperature-projection studies (Gosling et al., 2017), which is often conceptually linked to shifts in the location-specific minimum mortality temperature (Yin et al., 2019). Furthermore, while there is evidence of population adaptation to heat (Yin et al., 2019), limited evidence exists regarding human ability to adapt to cold (Arbuthnott et al., 2016). Existing research points to limits to physiological adaptation to heat and highlights the need of fundamental changes in urban planning, infrastructure design, and behavioral patterns, all of which are immensely complex and will take decades to achieve (Hanna and Tait, 2015).

Additionally, temperature projections were downscaled to a high resolution only from a single general circulation model (GCM), although an ensemble of GCMs would likely improve the accuracy of the estimated temperature-mortality burden. We limited the study design to only one model due to the computational burden of generating high-resolution model outputs for such long-term (decadal) simulations over the large spatial domains of Central and South America. Other studies showed that climate models tend to agree more closely on global mean temperature projections around mid-century than they do for projections toward the end of the century (Sillmann et al., 2013). Our study was also explicitly designed to quantify the upper and lower bounds of plausible scenarios and not to create a singular most accurate prediction. Next, city-level demographic projections were not available for our sample of Latin American cities, and we assumed that country-level patterns of change in the mortality rates and age distribution in 2045–2054 will apply to all cities within one country. We believe this is a reasonable assumption and an improvement on the existing literature for Latin America, given the lack of city-level data and the focus of the study to provide region-wide estimates of the temperature-mortality burden.

In conclusion, given expected changes in regional climate and demographics, the burden of mortality from both heat and cold is expected to increase in Latin American cities. Deaths from extreme heat will increase through the compounding effects of rising temperatures and expected demographic changes of an older population structure, lower mortality rates, and longer life expectancy that result in a larger population at elevated risk. While exposure to extreme cold will decrease because of climate change, population aging may counteract related decreases in cold-related mortality through an increasing population vulnerability to cold effects. Given these findings, we posit that public health officials and policy makers in Latin America should pay particular attention to the health needs of the older population when devising plans to protect the population from extreme temperatures. This includes designing climate adaptation policies that account not only for expected changes in ambient temperatures, but also for increasing population vulnerability to extreme temperatures. Heat action plans and long-term urban adaptations to reduce temperatures exposures and mitigate temperature-related health effects must be designed to be accessible to older adults, as well as individuals of all ages with limitations in physical or cognitive function (Kephart and Okoye, 2024).

## Supplementary Material

1

Appendix A. Supplementary material

Supplementary data to this article can be found online at https://doi.org/10.1016/j.envint.2025.109694.

## Figures and Tables

**Table 1:** City-level population, mortality, and temperature characteristics of 326 Latin American cities at baseline period of 2002–2015, median (10th, 90th percentiles).

	All countries	Argentina	Brazil	Central America^a^	Chile	Mexico	Peru
Number of cities	326	28	152	10	21	92	23
City population (thousands)^b^	267 (130, 1,292)	334 (133, 1,398)	221 (121, 1,292)	264 (185, 2,773)	210 (140, 920)	352 (142, 1,089)	288 (131, 885)
Population age distribution^b^							
% 0–49 years	81.2 (75.7, 85.8)	79.1 (73.9, 81.8)	79.3 (75.2, 84.9)	82.5 (79.4, 86.2)	76.4 (73.6, 79.9)	83.7 (81.0, 86.5)	83.8 (81.5, 86.5)
% 50–64 years	12.1 (9.3, 15.3)	13.1 (11.9, 14.5)	13.7 (10.1, 15.6)	10.7 (8.7, 13.1)	14.9 (13.8, 15.9)	10.6 (9.0, 12.2)	10.7 (9.1, 11.6)
% ≥ 65 years	6.7 (4.76, 9.1)	7.8 (6.7, 11.6)	7.1 (4.9, 9.1)	6.6 (5.1, 8.0)	8.8 (6.9, 10.7)	5.6 (4.2, 7.0)	5.5 (4.4, 6.8)
Annual deaths	1,454 (715, 6,825)	2,234 (821, 12,028)	1,443 (717, 6,279)	1,639 (1,085, 14,416)	1,088 (771, 5,215)	1,825 (775, 5,272)	978 (442, 3,644)
Mean temperature^c^	21.3 (14.9, 25.9)	17.5 (14.4, 21.6)	22.2 (18.9, 26.4)	23.8 (14.4, 25.8)	13.7 (10.8, 17.0)	20.3 (15.6, 25.8)	19.6 (8.0, 24.7)

a The Central American group in this analysis consists of cities in Guatemala, Panama, Costa Rica, and El Salvador.

b City population in 2010.

c Annual mean temperature.

**Table 2:** Temperature-related excess death fractions^1^ (A and B) and temperature-related death rates^2^ (C and D) under RCP2.6 and RCP8.5 emissions scenarios and projected changes in population size, age-specific mortality rates, and population age structure at mid-century in 326 Latin American cities.

	All countries	Argentina	Brazil	Central America*	Chile	Mexico	Peru
(A) Excess heat mortality (EDF, %)							
Baseline	0.87 (0.77; 0.96)^c^	1.32 (1.16; 1.48)	0.95 (0.80; 1.09)	0.38 (−0.13; 0.88)	0.08 (−0.07; 0.23)	0.83 (0.73; 0.93)	0.53 (0.21; 0.85)
RCP2.6	1.73 (1.51; 1.96)	2.45 (2.17; 2.73)	1.82 (1.60; 2.04)	0.01 (−0.58; 0.60)	−0.23 (−1.02; 0.57)	1.43 (1.23; 1.64)	6.62 (0.94; 12.29)
ΔRCP2.6-baseline	0.87 (0.63; 1.11)	1.13 (0.80; 1.45)	0.87 (0.61; 1.14)	−0.37 (−1.14; 0.41)	−0.30 (−1.11; 0.50)	0.60 (0.37; 0.83)	6.08 (0.40; 11.77)
RCP8.5	2.06 (1.80; 2.33)	2.77 (2.47; 3.08)	2.43 (2.07; 2.79)	−1.30 (−4.15; 1.55)	−0.22 (−1.27; 0.83)	2.07 (1.66; 2.49)	0.21 (−2.69; 3.11)
Δ RCP8.5-baseline	1.20 (0.92; 1.48)	1.45 (1.10; 1.80)	1.48 (1.09; 1.88)	−1.67 (−4.57; 1.22)	−0.30 (−1.36; 0.76)	1.24 (0.82; 1.67)	−0.32 (−3.24; 2.59)
(B) Excess cold mortality (EDF, %)							
Baseline	5.51 (5.26; 5.77)	10.41 (9.69; 11.12)	3.92 (3.61; 4.22)	4.40 (1.65; 7.16)	12.04 (9.95; 14.13)	6.48 (6.01; 6.95)	9.84 (8.48; 11.19)
RCP2.6	5.07 (4.80; 5.34)	10.21 (9.43; 11.00)	3.99 (3.70; 4.29)	3.75 (0.24; 7.25)	9.83 (8.15; 11.51)	5.84 (5.24; 6.44)	4.06 (3.13; 4.98)
Δ RCP2.6-baseline	−0.44 (−0.81; −0.07)	−0.19 (−1.26; 0.87)	0.08 (−0.35; 0.51)	−0.65 (−5.11; 3.80)	−2.21 (−4.89; 0.47)	−0.64 (−1.40; 0.12)	−5.78 (−7.42; −4.14)
RCP8.5	4.14 (3.90; 4.37)	9.35 (8.59; 10.10)	3.28 (3.02; 3.55)	2.37 (0.11; 4.64)	8.83 (7.19; 10.47)	4.39 (3.91; 4.88)	4.44 (3.28; 5.59)
Δ RCP8.5-baseline	−1.38 (−1.72; −1.03)	−1.06 (−2.10; −0.02)	−0.63 (−1.04; −0.23)	−2.03 (−5.59; 1.54)	−3.21 (−5.87; −0.55)	−2.09 (−2.76; −1.41)	−5.40 (−7.18; −3.62)
(C) Heat mortality rate (per 1,000)							
Baseline	0.53 (0.48; 0.59)	0.37 (0.32; 0.41)	0.78 (0.66; 0.9)	0.11 (−0.04; 0.26)	0.05 (−0.05; 0.15)	0.45 (0.4; 0.51)	0.15 (0.06; 0.24)
RCP2.6	1.74 (1.52; 1.96)	0.86 (0.77; 0.96)	2.44 (2.15; 2.74)	0.01 (−0.25; 0.26)	−0.23 (−1.06; 0.59)	1.45 (1.24; 1.66)	2.57 (0.36; 4.77)
Δ RCP2.6-baseline	1.21 (0.98; 1.44)	0.50 (0.39; 0.6)	1.66 (1.34; 1.98)	−0.11 (−0.4; 0.19)	−0.28 (−1.11; 0.54)	1.00 (0.78; 1.21)	2.42 (0.21; 4.62)
RCP8.5	2.07 (1.81; 2.34)	0.98 (0.87; 1.09)	3.26 (2.77; 3.75)	−0.56 (−1.80; 0.67)	−0.23 (−1.32; 0.86)	1.65 (1.23; 2.07)	−0.07 (−1.20; 1.06)
Δ RCP8.5-baseline	1.54 (1.27, 1.81)	0.61 (0.49; 0.73)	2.48 (1.97; 2.98)	−0.67 (−1.92; 0.57)	−0.23 (−1.38; 0.81)	2.10 (1.68; 2.52)	0.08 (−1.04; 1.21)
(D) Cold mortality rate (per 1,000)							
Baseline	3.39 (3.24; 3.55)	2.90 (2.7; 3.1)	3.24 (2.99; 3.49)	1.29 (0.48; 2.1)	7.80 (6.45; 9.16)	3.53 (3.27; 3.78)	2.79 (2.41; 3.18)
RCP2.6	5.10 (4.82; 5.37)	3.61 (3.33; 3.89)	5.36 (4.96; 5.76)	1.62 (0.11; 3.14)	10.21 (8.46; 11.96)	5.92 (5.31; 6.52)	1.57 (1.21; 1.93)
Δ RCP2.6-baseline	1.70 (1.39; 2.02)	0.71 (0.37; 1.05)	2.12 (1.64; 2.59)	0.33 (−1.39; 2.05)	2.41 (0.2; 4.62)	2.39 (1.73; 3.05)	−1.22 (−1.75; −0.69)
RCP8.5	4.15 (3.92; 4.39)	3.30 (3.04; 3.57)	4.40 (4.05; 4.76)	1.03 (0.05; 2.01)	9.17 (7.47; 10.87)	4.45 (3.96; 4.94)	1.72 (1.27; 2.17)
Δ RCP8.5-baseline	0.76 (0.48; 1.04)	0.40 (0.07; 0.74)	1.16 (0.73; 1.6)	−0.26 (−1.54; 1.01)	1.37 (−0.81; 3.54)	0.92 (0.37; 1.47)	−1.07 (−1.66; −0.48)

a Excess death fractions represent the percentages of deaths attributable to temperature (e.g., heat, cold) among all deaths in a population.

b Temperature-related mortality rates represent the number of deaths attributable to temperature (e.g., heat, cold) per 1,000 population.

c The parentheses contain 95% confidence intervals.

## Data Availability

Baseline city-specific temperature and mortality summaries and analysis outputs are freely available from an interactive app at https://drexel-uhc.shinyapps.io/MS85/. Links to the ERA5-Land, WorldPop and Global Urban Footprint source datasets used to estimate population-weighted ambient temperature, as well as final daily temperature outputs, are available at https://github.com/Drexel-UHC/salurbal_heat. Vital registration and population data for Brazil, Chile and Mexico were downloaded from publicly available repositories of statistical agencies in each country. Vital registration and population data for Argentina, Costa Rica, El Salvador, Guatemala, Panama and Peru were obtained directly from statistical agencies in each country. A link to these agency websites can be accessed via https://drexel.edu/lac/data-evidence/data-acknowledgements.

## Code availability:

The code to reproduce the main analysis is available at https://github.com/mariabak1/Temperature-mortality-projections.
