# Supplementary material for: Future temperature-related mortality in Latin American cities under climate change and population scenarios
Source: Environ Int. Author manuscript; Available in PMC 2025 Sep 26. (PMC12467326; doi:10.1016/j.envint.2025.109694)

**Supplemental materials**

**Supplemental Table S1.** Projected changes in temperature under RCP2.6 and RCP8.5 emissions scenarios from 2002-2015 to 2045-2054 periods in 326 Latin American cities, presented as median (25^th^, 75^th^ percentiles).

| **Annual mean  temperature °C** | **All countries** | **Argentina** | **Brazil** | **Central America*** | **Chile** | **Mexico** | **Peru** |
| --- | --- | --- | --- | --- | --- | --- | --- |
| Baseline: temperatures | 20.9 (17.8, 23.7) | 16.8 (15.4, 18.6) | 22.0 (20.1, 23.9) | 23.6 (20.1, 25. 3) | 13.7 (12.3, 14.7) | 20.3 (17.2, 24.3) | 19.5 (11.6, 22.5) |
| RCP2.6: temperatures | 21.9 (18.9, 24.6) | 18.5 (17.0, 20.1) | 23.0 (20.9, 24.7) | 24.5 (21.2, 26.4) | 15.0 (13.2, 16.1) | 21.2 (18.3, 25.6) | 22.9 (12.5, 25.1) |
| RCP2.6: delta from baseline | 1.0 (0.8, 1.2) | 1.7 (1.5, 1.8) | 0.9 (0.8, 1.0) | 1.0 (0.9, 1.1) | 1.3 (1.1, 1.9) | 0.9 (0.6, 1.3) | 1.5 (1.1, 3.0) |
| RCP8.5: temperatures | 22.4 (19.6, 25.2) | 18.9 (17.5, 20.4) | 23.6 (21.5, 25.4) | 25.6 (22.0, 27.2) | 15.6 (14.0, 16.6) | 22.3 (19.4, 26.0) | 21.5 (13.5, 24.5) |
| RCP8.5: delta from baseline | 1.7 (1.4, 2.0) | 2.0 (1.8, 2.2) | 1.5 (1.2, 1.7) | 1.9 (1.8, 1.9) | 1.8 (1.5, 2.1) | 1.9 (1.6, 2.2) | 2.0 (1.8, 2.2) |

**Supplemental Table S2**. Population size, age-specific mortality rates, and age structure at baseline (2002-2015) and mid-century (2045-2054) across the Latin American countries in the study

| **Demographic indicator** | **All countries** | **Argentina** | **Brazil** | **Central America*** | **Chile** | **Mexico** | **Peru** |  |
| --- | --- | --- | --- | --- | --- | --- | --- | --- |
|  |  |  |  |  |  |  |  |  |
| Total population (in 1,000s) |  |  |  |  |  |  |  |  |
| Total population at baseline | 424,503 | 41,961 | 193,307 | 29,908 | 16,926 | 112,728 | 29,673 |  |
| Total population at mid-century | 530,960 | 51,522 | 230,767 | 42,539 | 20,663 | 143,579 | 41,891 |  |
|  |  |  |  |  |  |  |  |  |
| Age-specific mortality rates (ASMR) per 1,000 people | | |  |  |  |  |  |  |
| ASMR 0-49 at baseline | | 0.009 | 0.006 | 0.010 | 0.010 | 0.005 | 0.009 | 0.008 |
| ASMR 0-49 in 2045-2054 | | 0.004 | 0.003 | 0.004 | 0.005 | 0.002 | 0.005 | 0.004 |
| ASMR 50-64 at baseline | | 0.043 | 0.045 | 0.048 | 0.043 | 0.034 | 0.049 | 0.042 |
| ASMR 50-64 in 2045-2054 | | 0.025 | 0.024 | 0.023 | 0.027 | 0.016 | 0.030 | 0.021 |
| ASMR 65+ at baseline | | 0.467 | 0.444 | 0.532 | 0.455 | 0.438 | 0.449 | 0.522 |
| ASMR 65+ in 2045-2054 | | 0.401 | 0.386 | 0.438 | 0.392 | 0.350 | 0.428 | 0.439 |
|  |  |  |  |  |  |  |  |  |
| Population age distribution |  |  |  |  |  |  |  |  |
| % 0-49 at baseline | 81 | 75 | 81 | 82 | 75 | 83 | 81 |  |
| % 0-49 in 2045-2045 | 62 | 62 | 58 | 65 | 53 | 62 | 66 |  |
| % 50-64 at baseline | 12 | 14 | 13 | 11 | 15 | 11 | 11 |  |
| % 50-64 in 2045-2054 | 19 | 19 | 21 | 19 | 21 | 19 | 18 |  |
| % 65+ at baseline | 7 | 11 | 7 | 7 | 10 | 6 | 7 |  |
| % 65+ in 2045-2054 | 19 | 19 | 22 | 16 | 26 | 19 | 16 |  |

* Central America includes cities in El Salvador, Guatemala, Costa Rica, and Panama

**Supplemental Table S3**. Baseline and projected age-specific relative risk (RR) of mortality (95% CI in parentheses)

|  |  | RCP2.6 | | | RCP8.5 | | |
| --- | --- | --- | --- | --- | --- | --- | --- |
|  |  | 0-49 | 50-64 | 65+ | 0-49 | 50-64 | 65+ |
| RR at 95^th^ percentile vs MMT* | Baseline | 1.053  (1.048,1.058) | 1.013  (1.010,1.015) | 1.063  (1.059,1.067) | 1.053  (1.048,1.058) | 1.013  (1.010,1.015) | 1.063  (1.059,1.067) |
|  | Projected | 1.073  (1.065,1.080) | 1.005 (1.003,1.007) | 1.131  (1.119,1.143) | 1.097  (1.087,1.108) | 1.001  (0.998,1.004) | 1.189  (1.172,1.206) |
| RR per 1°C increase over 95^th^ percentile temperature | Baseline | 1.014  (1.005,1.023) | 0.989  (0.980,0.998) | 1.051  (1.042,1.060) | 1.014  (1.005,1.023) | 0.989  (0.980,0.998) | 1.051  (1.042,1.060) |
|  | Projected | 1.020  (1.006,1.035) | 0.983  (0.966,0.998) | 1.058  (1.042,1.074) | 1.028  (1.010,1.047) | 0.997  (0.977,1.017) | 1.087  (1.067,1.108) |

*MMT is the minimum mortality temperature, or the temperature at which the risk of mortality is the lowest.

The percentiles refer to the city-specific daily temperature distribution during the baseline and projection periods.

**Supplemental Figure S1**. City-level heat-related EDF at baseline period (A) with projected changes in annual temperature and population demographics (population size, age-specific mortality rates, and population age structure) from baseline period to 2045-2054 under RCP2.6 (B) and RCP8.5(C) greenhouse gas emissions scenarios.


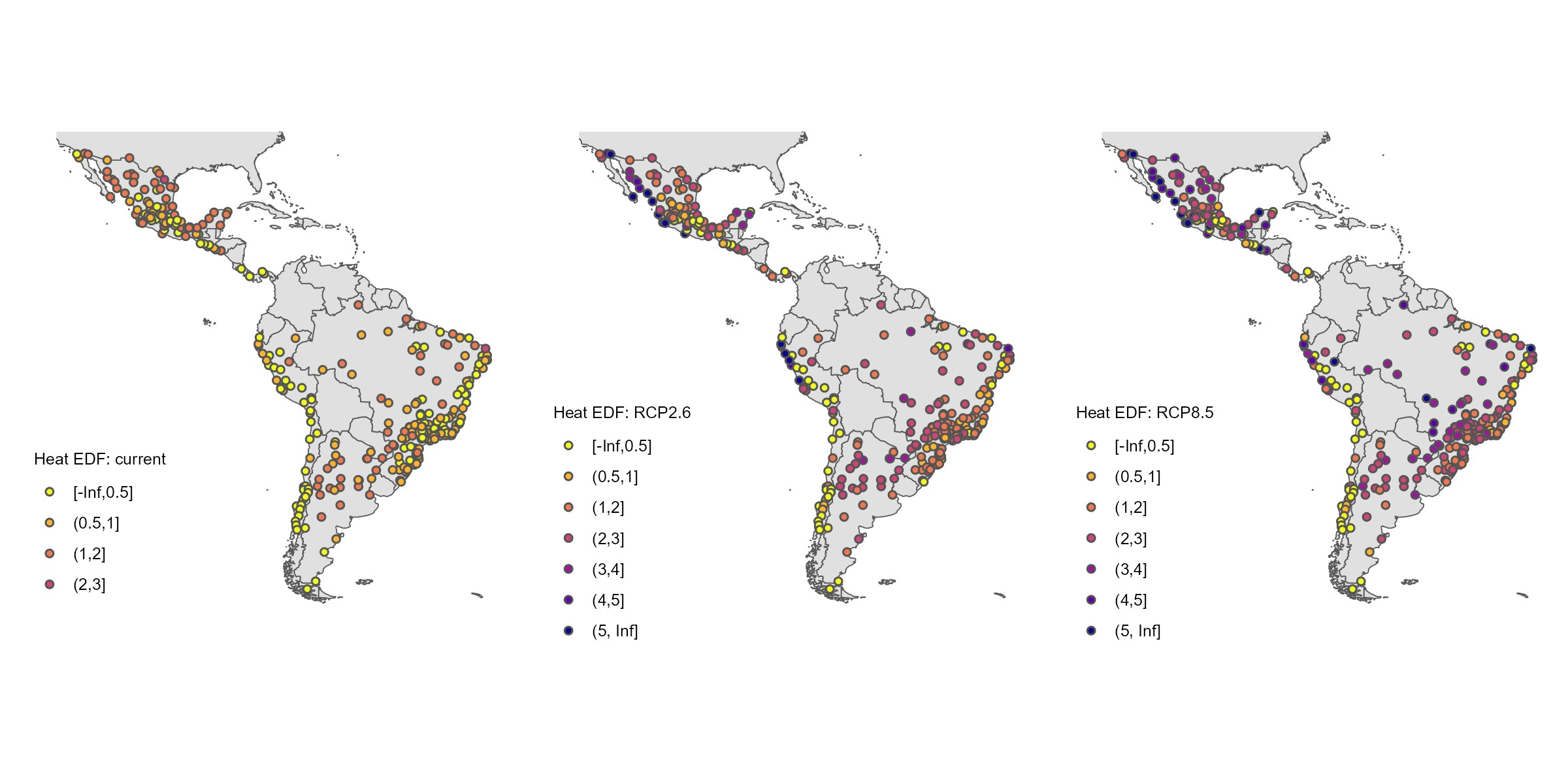


**Supplemental Figure S2**. City-level cold-related EDF at baseline period (A) with projected changes in annual temperature and population demographics (population size, age-specific mortality rates, and population age structure) from baseline period to 2045-2054 under RCP2.6 (B) and RCP8.5(C) greenhouse gas emissions scenarios.


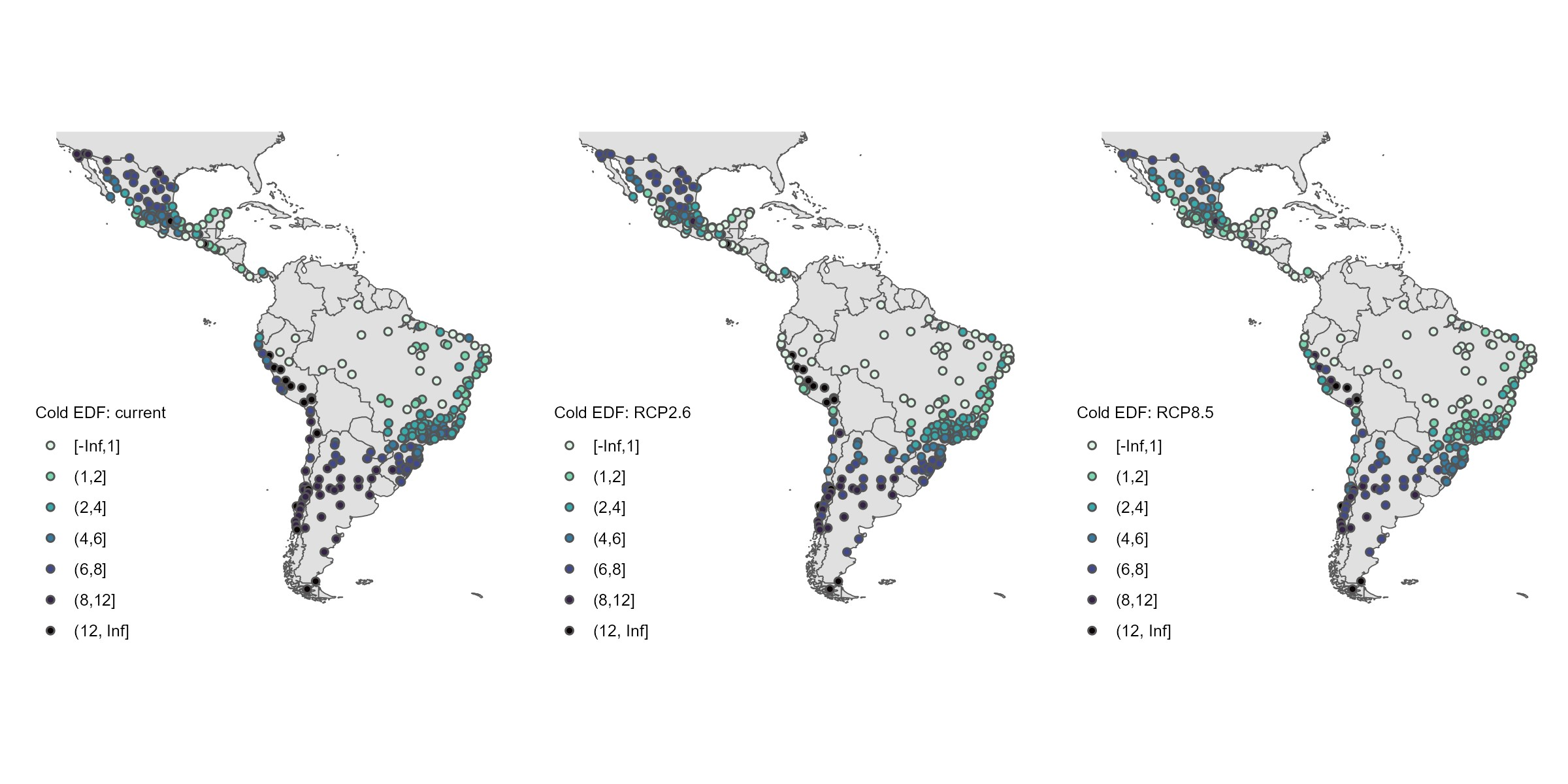


**Supplemental Figure S3**. City-level heat-related mortality rate (annual deaths per 1,000 residents) at baseline period (A) with projected changes in temperature and demographics (population size, age-specific mortality rates, and population age structure) from baseline period to 2045-2054 under RCP2.6 (B) and RCP8.5(C) greenhouse gas emissions scenarios.


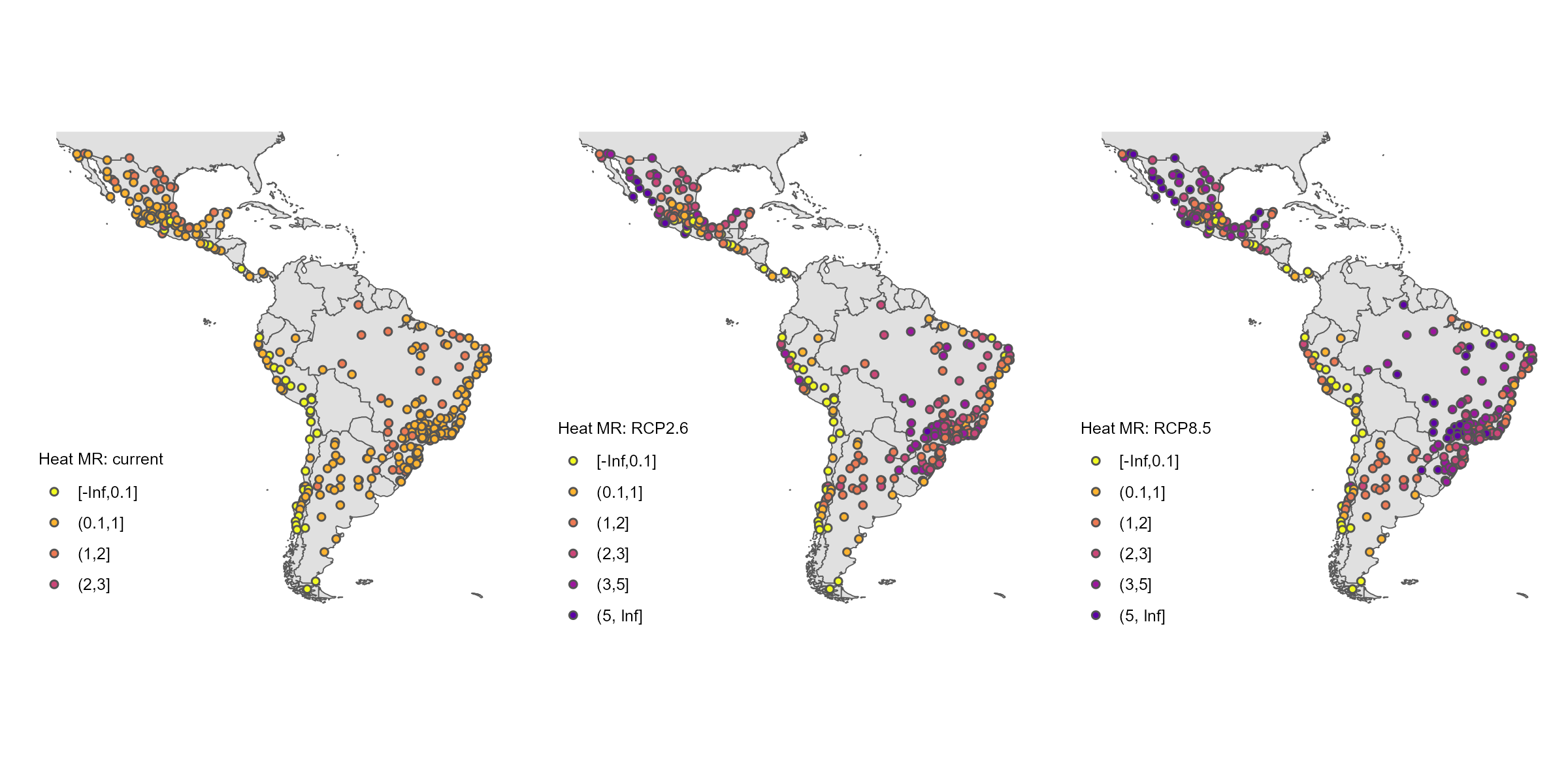


**Supplemental Figure S4**. City-level cold-related mortality rate (annual deaths per 1,000 residents) at baseline period (A) with projected changes in temperature and demographics (population size, age-specific mortality rates, and population age structure) from baseline period to 2045-2054 under RCP2.6 (B) and RCP8.5(C) greenhouse gas emissions scenarios.


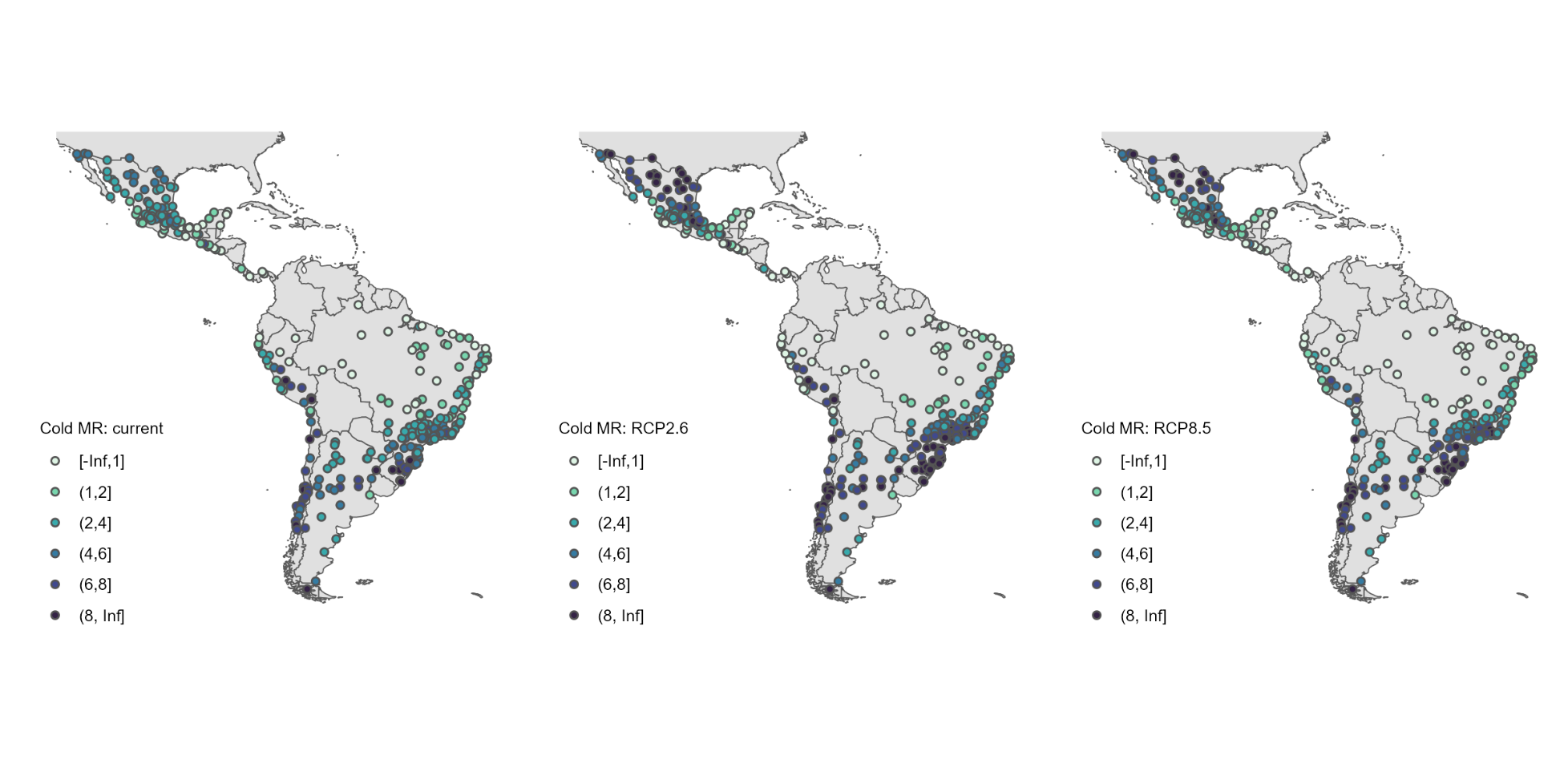
**Supplemental Figure S5**. Country-level heat-related EDFs at baseline period with projected changes in mean daily temperature from baseline period to 2045-2054 under RCP2.6 and RCP8.5 greenhouse gas emissions scenarios and mid-century changes in the population size, age-specific mortality rates, and age structure.


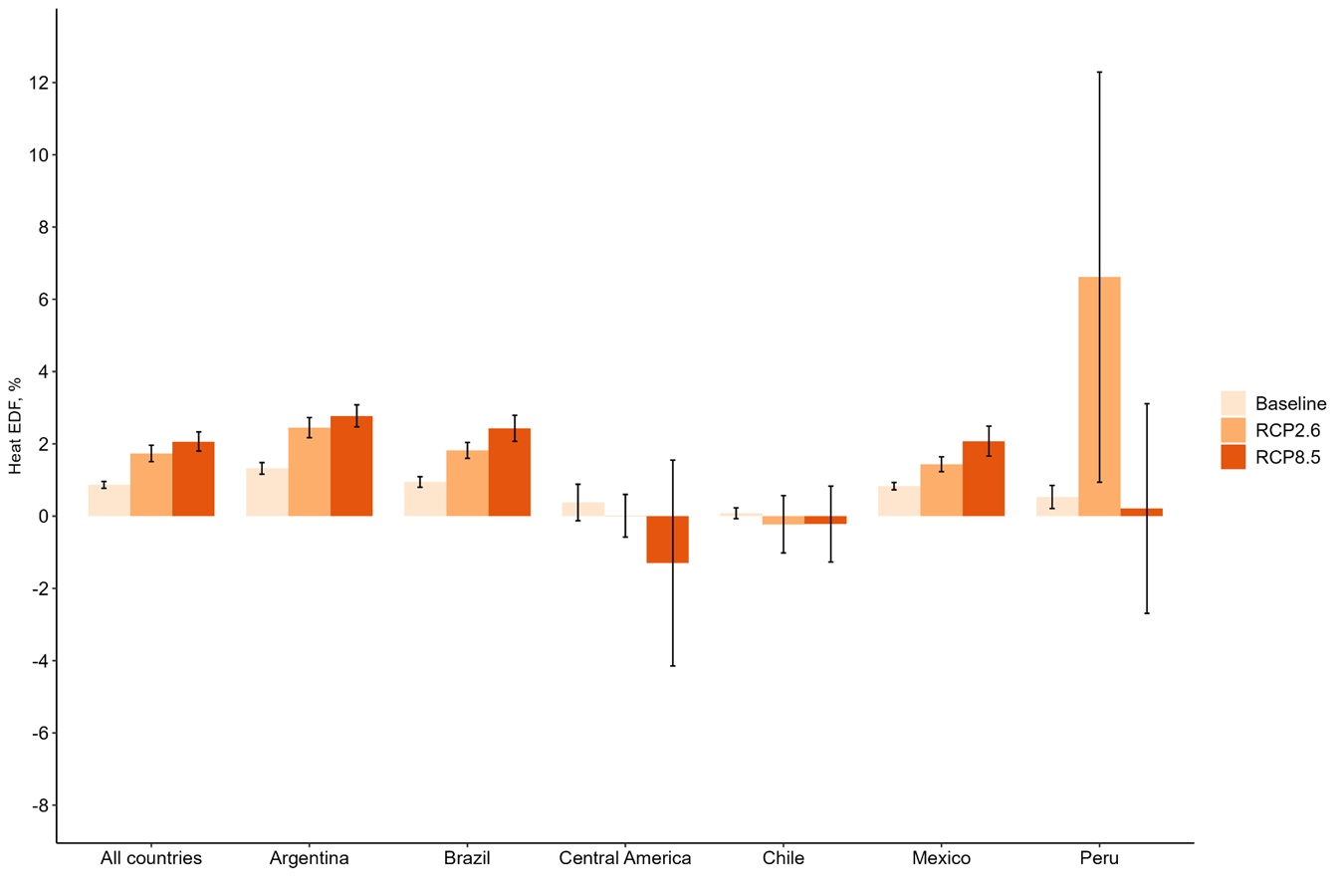


**Supplemental Figure S6**. Country-level cold-related EDFs at baseline period with projected changes in mean daily temperature from baseline period to 2045-2054 under RCP2.6 and RCP8.5 greenhouse gas emissions scenarios and mid-century changes in the population size, age-specific mortality rates, and population age structure.


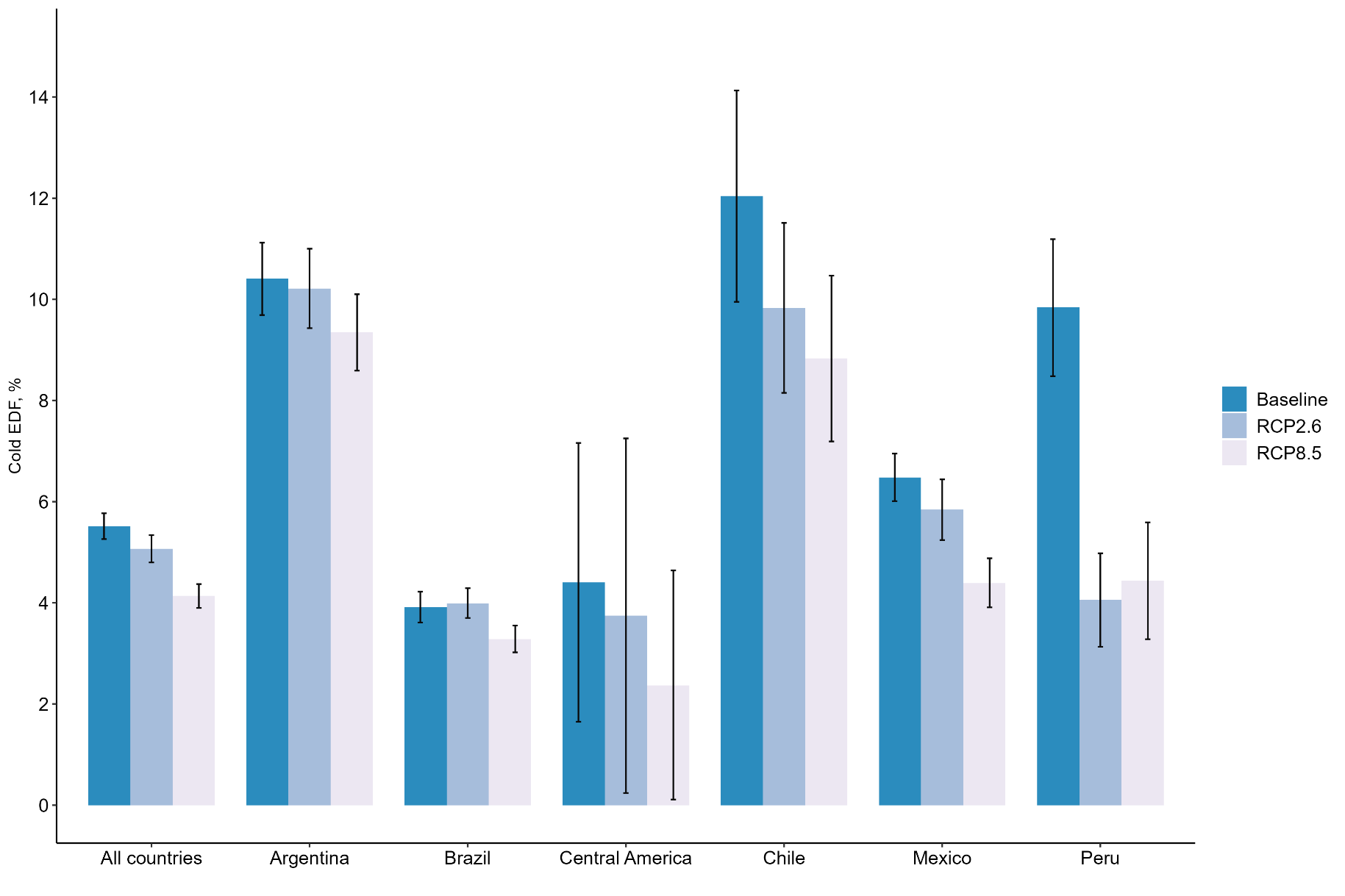


**Supplemental Figure S7**. Country-level heat-related mortality rates at baseline period with projected changes in mean daily temperature from baseline period to 2045-2054 under RCP2.6 and RCP8.5 greenhouse gas emissions scenarios and mid-century changes in the population size, age-specific mortality rates, and age structure.


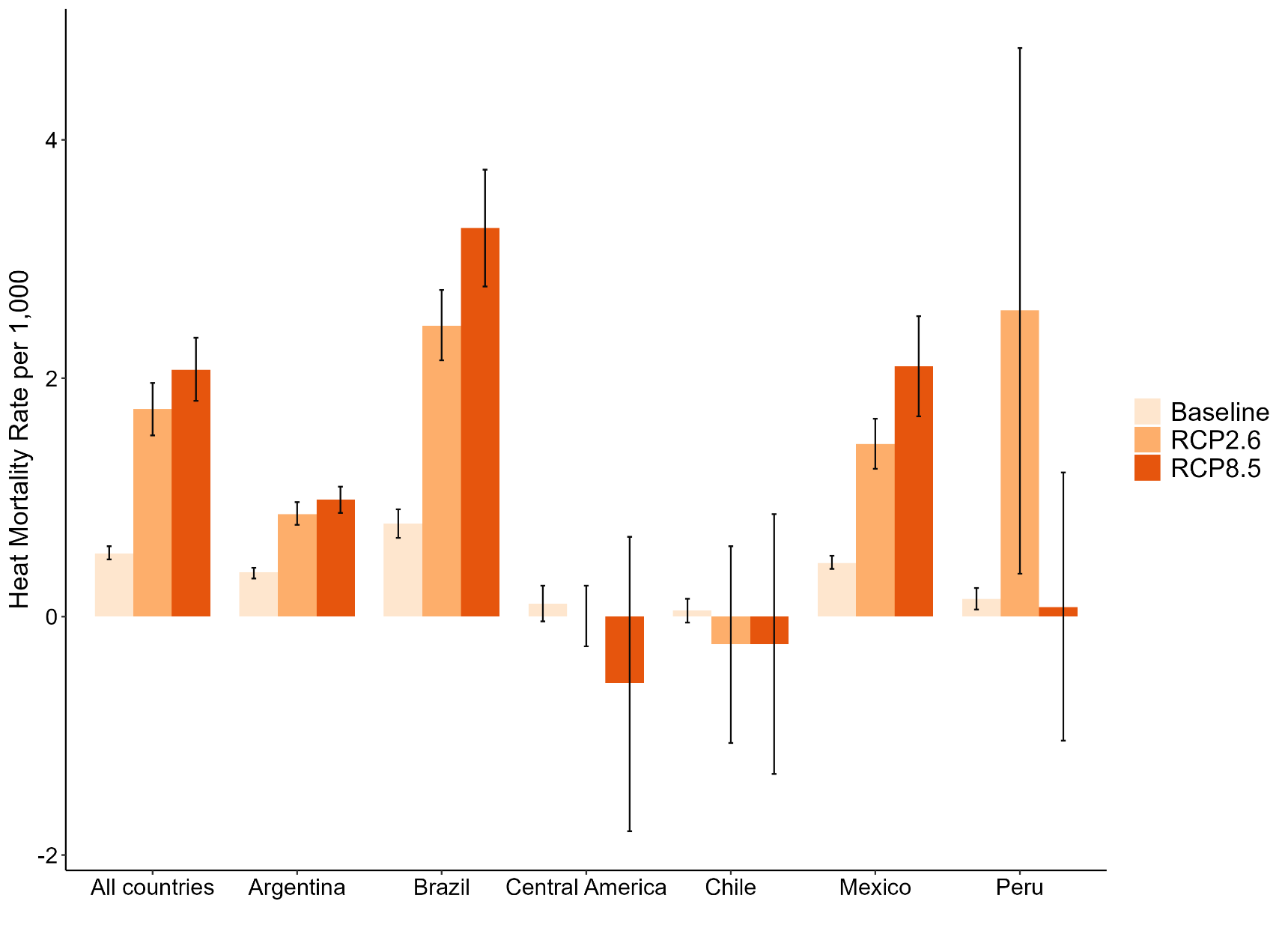


**Supplemental Figure S8**. Country-level cold-related mortality rates at baseline period with projected changes in mean daily temperature from baseline period to 2045-2054 under RCP2.6 and RCP8.5 greenhouse gas emissions scenarios and mid-century changes in the population size, age-specific mortality rates, and age structure.


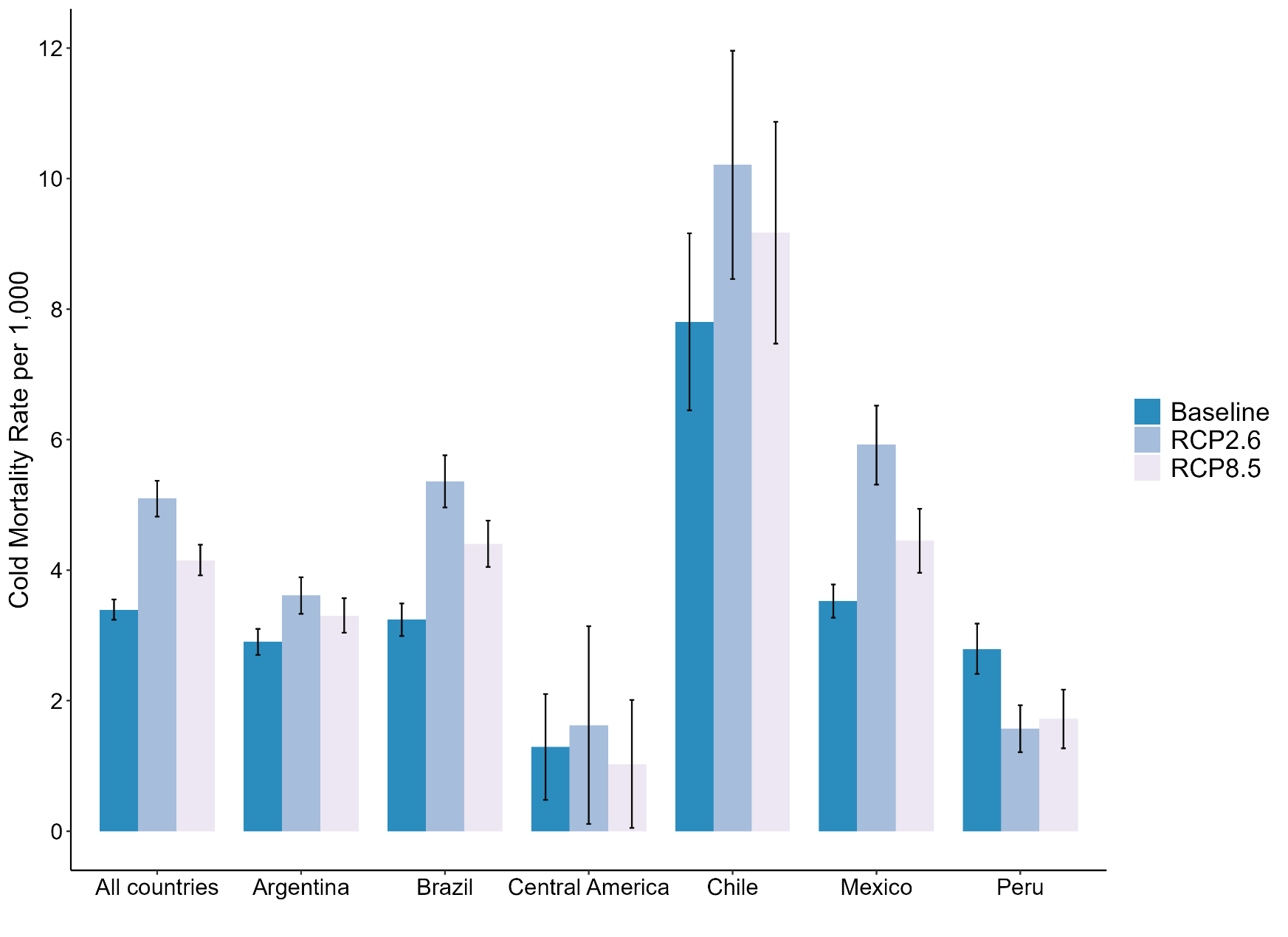

Supplement: 1 [file NIHMS2105997-supplement-1.docx]
